# Enhancing emergency response in Somalia: Evaluating the WHO Mass Casualty Management course in trauma hospitals

**DOI:** 10.1016/j.afjem.2026.100948

**Published:** 2026-02-05

**Authors:** Mohamed Mukhtar Ali, Barkhad Mohamed Ismail, Mohamed Hussein, Meaghan M. Sydlowski, Nichole Michaeli

**Affiliations:** a University of Hargeisa, Hargeisa, Somaliland, Somalia; b General physician, Hargeisa Group Hospital, Gacan libah district, Hargeisa, Somaliland, Somalia; c International Medical Corps, Mogadishu, Somalia; d Emergency Response Specialist, International Medical Corps, Washington, D.C., USA; e Department of Emergency Medicine, University of Vermont, USA

**Keywords:** Emergency medicine training, Emergency care, Mass casualty management, Preparedness, Emergency units

## Abstract

**Introduction:**

The ongoing conflict between the Somali National Army and non-state armed actors has led to an increase in mass casualty incidents (MCIs) which burden the fragile Somali healthcare system. Hospitals often become overwhelmed during MCIs, which can lead to increased mortality. To address this gap in mass casualty management (MCM), the WHO developed the MCM course to help hospitals address MCIs through the creation of individualized MCM plans. This study evaluated the effect of the WHO MCM course on participants’ knowledge of MCM concepts and self-reported confidence in managing MCIs in trauma hospitals in Somalia.

**Methods:**

A prospective pre-post educational study was conducted with healthcare teams from trauma hospitals across Somalia in October 2023. The four-day MCM course utilized lectures and interactive tabletop exercises to introduce MCM concepts, simulate MCI scenarios, and help participants develop MCM plans. Participants’ knowledge was assessed using multiple choice pre- and post-tests. Confidence was evaluated using a 4-point Likert scale surveys, feedback forms were used to assess course acceptability. Paired t-tests and Cohen’s d were used to analyze pre-post changes.

**Results:**

The MCM course was completed by 23 participants from 3 regions across Somalia. They had an average of 8.5 years of clinical experience and 3 years of MCM experience. The mean post-course test scores (78.9%) showed a significant improvement (p<0.001; Cohen's d=1.82) compared to pre-course test scores 47.9% (28%-72%). There was also an improvement in self-reported confidence in the implementation and activation of MCM plans (d=0.49), understanding of MCM roles and responsibilities (d=0.55). The largest gains were in operational readiness (d=1.42), including clearing the emergency unit, documentation, and triage planning.

**Conclusion:**

The MCM course improved participant’s knowledge of key MCM concepts and self-reported confidence in managing MCIs. Although limited by small sample size and reliance on self-reported outcomes, findings suggest the course may support improved MCM readiness in conflict-affected settings.

## African relevance

• Conflict and mass casualty incidents (MCIs) strain healthcare in Somalia and Africa
• This study highlights the need for mass causualty management (MCM) training in resource-limited settings
• Armed conflicts and MCIs expose the gaps in Africa’s healthcare systems
• This study highlights the need for hospital-specific MCM plans in Africa
• Somalia’s MCM success shows the WHO MCM course can aid African nations

## Introduction

Over the past three decades, Somalia has faced a surge in conflict, including ongoing clashes between the Somalia National Army and non-state armed groups. This instability has led to a significant rise in mass casualty incidents (MCIs), events where the number of casualties overwhelms available resources. From January to October 2020, MCIs increased by 30% at four major International Committee of the Red Cross (ICRC) supported hospitals: Medina and Keysaney Hospitals in Mogadishu, Bay Hospital in Baidoa, and Kismayo Hospital in Kismayo [1]. One of the most devastating events occurred on 14 October 2017, when twin truck bombings in Mogadishu killed 587 people and injured nearly 1,000 others, marking the deadliest attack in Somalia’s history and the second deadliest terrorist incident in Africa [2]. A 2021 World Health Organization (WHO) assessment further highlighted the burden of trauma on Somalia’s health system, reporting over 37,000 civilian trauma cases in 136 hospitals in a single year, largely due to conflict-related injuries [3]. In 2022 alone, 57 conflict-related MCIs were reported in ICRC-supported hospitals between January and October, up from 43 during the same period in 2021 [1].

Hospital based emergency units often represent the frontline of the health system during MCIs and are key to an effective emergency response. In Somalia, however, healthcare staff are frequently underprepared and lack access to essential tools and equipment, limiting their ability to respond to MCIs and contributing to preventable disability and death [4]. When emergency units are overwhelmed, both direct mortality from the acute event and preventable mortality from everyday conditions increase dramatically.

This challenge is not unique to Somalia. A systematic review of hospital disaster preparedness across Sub-Saharan Africa revealed major deficiencies in training, infrastructure, and emergency coordination. Many hospitals lacked comprehensive emergency preparedness plans, skilled personnel, and sufficient resources to respond to large-scale disasters effectively [5]. Similarly, a study assessing emergency and disaster preparedness in public hospitals in Ethiopia reported inadequate emergency and disaster preparedness, with significant gaps in operational systems, communication protocols, and logistics [6]. These findings are consistent with broader global analyses showing that trauma systems and hospitals in low and middle income countries (LMICs) are often under-resourced for mass casualty preparedness [7].

At the same time, evidence on disaster and mass-casualty training for healthcare workers remains limited and heterogeneous. Systematic reviews have found that training interventions can improve knowledge and confidence, but most studies come from high-income or non-conflict settings, often with small samples and diverse outcome measures [8]. More recent work describes simulation-based mass-casualty training for medical students and residents, demonstrating improvements in knowledge and self-reported preparedness [[9], [10], [11], [12]]. Extended-reality and virtual approaches to MCI training are also emerging, largely focused on pre-hospital responders in high-resource settings [13]. However, hospital-based mass casualty management (MCM) training in fragile and conflict-affected contexts remains under-evaluated.

To address the systemic gaps in mass casualty management, the WHO established minimum standards for MCM at the level of the emergency unit and developed the Mass Casualty Management (MCM) course to help hospitals meet these standards [14,15]. The MCM course has been taught in multiple regions and countries including Somalia, Iran, the Gaza Strip, the West Bank, Lebanon, Ethiopia, Ukraine, and Moldova [4,[16], [17], [18], [19], [20]]. Despite its broad adoption and relevance to crisis-affected settings, there are no published studies evaluating the MCM course in regions experiencing active conflict.

In response to the ongoing conflict in Somalia, International Medical Corps (IMC), initiated a collaborative effort between its Somalia country mission and headquarters’ Emergency Response Unit (ERU) to assess existing training needs and identify targeted interventions to strengthen emergency preparedness and response. IMC, an international NGO with a long-standing focus on emergency relief in conflict and disaster settings, has implemented a comprehensive package of health interventions in Somalia since 1991. Currently operating in Mogadishu, Jowhar, Galkayo, and Baidoa, IMC’s programs aim to improve the availability, accessibility, and utilization of health services for vulnerable populations and strengthen Somalia’s fragile health system [21]. Based on IMC’s assessments, the MCM course was identified as an intervention to strengthen mass casualty preparedness at regional health centers and hospitals.

This study aimed to evaluate the effect of the WHO MCM course on participants’ knowledge of MCM concepts and self-reported confidence in managing mass casualty incidents in trauma hospitals in Somalia. This evaluation focuses on short-term educational outcomes among frontline healthcare providers working in a fragile, conflict-affected setting.

## Methods

### Study design and setting

This was a prospective, single-group pre-post educational evaluation of the WHO MCM course delivered to emergency care providers from major public hospitals and primary healthcare facilities in Somalia in October 2023. The course was hosted at a trauma hospital in Mogadishu due to security constraints in other regions.

### Participants and recruitment

The MCM course was offered to emergency department healthcare providers from the largest public hospitals in Somalia, with a focus on hospitals in major cities. Engagement was initiated with hospital management, including administrators and directors of emergency departments. Formal invitations were then extended to the selected hospitals. Department heads and team leaders nominated staff members, including managers, nurses, surgeons, and other clinicians with roles in emergency care and mass-casualty response.

Twenty-four participants attended the course from eight hospitals and healthcare facilities. Participants included 14 doctors and 10 nurses and managers. For quantitative analysis, we included only participants who completed both pre-and post-course assessments (n=23). One participant who completed only post-course tools was excluded from paired analysis. Participants’ demographic data (age, gender, years of clinical experience, and years of MCM experience) were collected at baseline. Years of MCM experience were self-reported and defined as the number of years participants had been involved in any role related to mass casualty management (e.g., participation in MCIs, membership of hospital disaster planning committees, or designated MCM responsibilities).

### Course description

The MCM course was conducted over a four day period. The morning sessions focused on MCM concepts, group discussions, and exercises. The afternoon sessions focused on the tabletop exercise where facilitators challenged teams to practice the concepts introduced in the morning sessions with MCI scenarios. The tabletop exercises encouraged hospital teams to develop plans and find operational solutions to simulated problems. The roles and coordination of the healthcare workers (doctors, nurses) and non-health staff (administrative staff, stretcher bearers, security, volunteers) involved in MCM were included in the scenarios. Participants were asked to play their own role in the exercise using magnets and their own hospital blueprint.

On day four, participants wrote their MCM plans using checklists and presented their plans to the instructors. The MCM plan was the central deliverable of the course, developed collaboratively by course participants and intended for direct application within their hospital settings. This is a structured, emergency department-specific protocol designed to guide emergency response during a MCI. It includes procedures for triage, patient flow, communication, documentation, and resource allocation during high-casualty events. Debriefing sessions identified and addressed gaps, challenges and recommendations for improvements. Although development of an MCM plan was a central educational deliverable, plans were not formally scored or analyzed in this study.

### Assessment tools and data analysis

Participants’ knowledge and confidence in MCM was assessed with standardized versions of the pre- and post-course tests and surveys. These tools are part of the WHO MCM course’s established evaluation framework and were administered without modification. The tests and surveys were reviewed by Somali and international emergency care clinicians to ensure clarity. The pre- and post-course tests consisted of multiple choice questions, while the pre- and post-course surveys assessed participants’ confidence in managing MCIs using a 4-point Likert scale. Survey items were grouped into three domains to facilitate analysis: clinical confidence, which captured participants’ self-reported comfort managing injured patients; understanding of roles and MCM plans, which assessed knowledge of team responsibilities and incident management structure; and operational readiness, which evaluated perceptions of preparedness, coordination, and the ability to implement MCM procedures during an actual event. Qualitative data from the MCM feedback form were analyzed to identify common patterns and insights. The assessment tools were administered in English, and Somali-speaking instructors provided real-time clarification as needed.

We summarized continuous variables using means and ranges and categorical variables using frequencies and percentages. Survey responses were treated as continuous variables on a 4-point Likert scale. Pre- and post-course scores were then compared using paired t-test. To quantify the magnitude of change, Cohen’s d effect sizes were calculated from pre- and post-course tests and survey and were analyzed using the paired t-test. The data were analyzed using STATA/SE 18.0.

### Ethical considerations

This study was reviewed by the Mass General Brigham Institutional Review Board and Research and Ethical Committee at the Somali Ministry of Health (Ref/MOHHS/DGO/0871/Nov2023). The Mass General Brigham Institutional Review Board and Research provided an IRB waiver, since the data collected were limited to the standardized assessment tools, which are part of the WHO MCM training package. These instruments are routinely used for course evaluation and quality improvement.

## Results

**Fig. 1:**
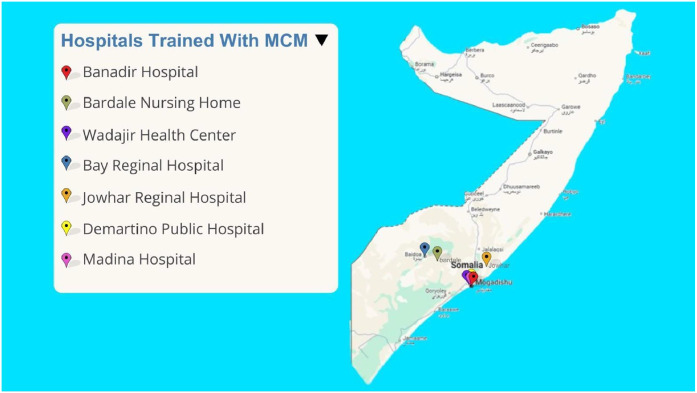
Map of Somalia with location of hospitals and health center of MCM participants.

The assessment included 23 participants (14 doctors and 8 nurses) from three regions and eight hospitals and healthcare facilities across Somalia (Fig. 1). Participants had an average of 8.5 years of clinical experience (range 4-20 years) and three years of mass casualty management experience (range: 0-15 years). The course included a mix of early to mid-career health care professionals with an average age of 34 years (range: 25 to 50 years). Seventy-eight percent of participants were male and 22 percent were female (Table 1).

**Table 1:** Demographic information.

**Demographic data**				

**Variable**	**Observations**	**Mean**	**Min**	**Max**

Age	23	34	25	50
Years of clinical experience	23	8.5	4	20
Years of mcm experience	23	3	0	15

**Gender**	**Frequency**	**Percent**		

Male	18	78%		
Female	5	22%		

Twenty-three participants completed both pre- and post-course assessments and were included in the analysis (one participant was not present for the pre-test). The mean post-course test scores (78.9 %) showed a significant improvement (p < 0.001) compared to pre-course test scores (mean 47.9%) with a very large effect size (d=1.82) (Table 2).

**Table 2:** Knowledge test scores.

Pre-Test Mean (Range) n=23	Post-Test Mean (Range) n=23	Paired T-test	Cohen’s d
47.9% (28-72)	78.9% (56-98)	p<0.001	1.82

The MCM pre-course survey was completed by 23 participants, while the MCM post- course survey was completed by 24 participants (one participant was not present for the pre-test). The post-course survey of the participant who did not complete the pre-course survey was excluded from the final data analysis.

Following the MCM course, there was a significant increase in self-reported confidence in the implementation and activation of MCM plans. Additionally, participants reported increased knowledge of MCM roles and responsibilities, ability to triage and track patients during an MCI, and understanding of the supplies needed in the red and green zones. Interestingly, participants report significantly lower confidence in their colleagues’ understanding of the hospital MCM plan after taking the course (Table 3). Freetext survey responses were analysed thematically with common themes identified and illustrative quotes presented in Table 4.

**Table 3:** Domain-level and subgroup analysis of pre–post survey results (n = 23).

Group	Domain / Outcome	Pre Mean (SD)	Post Mean (SD)	Mean Difference	Cohen’s d	p-value
**Overall (n = 23)**	**Clinical confidence**	3.17 (0.54)	3.56 (0.36)	0.39	0.68	<0.01
	**Understanding of plans & roles**	3.06 (0.48)	3.27 (0.43)	0.2	0.35	0.04
	**Operational readiness**	2.71 (0.62)	3.57 (0.41)	0.85	1.42	<0.001
**Clinical experience ≤5 years (n = 6)**	Overall confidence	2.73	3.25	0.52	1.46	<0.01
	Operational readiness	2.5	3.17	0.67	1.14	0.005
**Clinical experience >5 years (n = 17)**	Overall confidence	3.08	3.51	0.43	0.87	<0.01
	Operational readiness	2.79	3.71	0.92	1.51	<0.001
**No prior MCM experience (n = 6)**	Overall confidence	3	3.23	0.22	0.41	0.32
	Operational readiness	2.72	3.47	0.75	1.18	0.004
**Any prior MCM experience (n = 17)**	**Overall confidence**	**2.98**	**3.52**	**0.53**	**1.31**	**<0.001**
	**Operational readiness**	**2.71**	**3.6**	**0.89**	**1.48**	**<0.001**

**Table 4:** Participant feedback on course quality (n = 24).

Common Themes	Short summary of comment themes	Quotes
Course Effectiveness	Course content was seen as highly relevant, clear, and directly applicable to real MCI scenarios and daily work.	“I now understand the best way to manage MCIs”; “Will apply in my hospital”; “Helpful for our hospital”; “Applicable to MCIs”
Knowledge and Skills	Participants reported substantial gains in MCM knowledge, confidence, and practical skills.	“Expanded my knowledge”; “Very informative”, “I feel confident to work in MCI management”
Suggested Changes	Participants requested additional materials, equipment, and more frequent trainings to support sustained implementation.	“Need equipment for training colleagues”; “We need this every 3 or 6 months”;

## Discussion

This study evaluated the WHO MCM course delivered in an active conflict setting and demonstrated substantial improvements in participants’ knowledge and self-reported confidence. The magnitude of improvement in operational readiness was particularly notable, with very large effect sizes across tasks essential to MCI response, such as clearing the emergency unit, supplying treatment zones, documentation, and triage.

Participants also gained confidence in activating their hospital’s MCM plan and executing their assigned roles, despite ongoing system-level weaknesses. Notably, participants reported decreased confidence in colleagues’ understanding of MCM plans, suggesting broader institutional training gaps beyond individual capacity building. Combined domain-level and subgroup analyses of the survey results showed significant improvements across all participant groups, with particularly large gains in operational readiness (overall d = 1.42), early-career clinicians (d = 1.46), and participants with prior MCM experience (d = 1.48). This suggests that the course may reinforce existing knowledge and provide structured frameworks that support more experienced staff.

Participant feedback further supported the acceptability and relevance of the course. All participants reported that the teaching was clear, relevant, and improved their knowledge, skills, and confidence, and nearly all felt it was well tailored to the Somali context. Qualitative comments highlighted strong satisfaction with the course, perceived applicability to real MCI situations, and requests for additional training, materials, and equipment. These findings reinforce the value of the MCM course as both feasible and well-received in a conflict-affected setting.

Overall, our findings align with previous research demonstrating that structured MCI training can improve provider preparedness [11]. Our results expand this evidence base by showing feasibility and impact of the WHO MCM course in a conflict-affected LMIC context where evidence is limited. To our knowledge, this is the first time the WHO MCM course has been formally evaluated in the peer-reviewed literature. By providing data on course outcomes, this study fills an important gap and establishes a foundation for future studies in similar settings. MCM courses are particularly relevant to regions facing active conflict, but there are important considerations when conducting training as attacks on healthcare facilities and personnel have increased significantly in recent years [22].

This MCM training was held at a trauma hospital in Mogadishu due to safety and security concerns from the active conflict in Jowhar, Galkayo, and Baidoa. The centralized training location limited participant availability, so the training team focused on ensuring that attendees had the knowledge, skills, and supplies to teach and implement the MCM plans at their respective hospitals. Future trainings may be able to broaden participation by integrating virtual learning options for those unable to travel safely.

While MCIs are ideally managed at large referral and trauma hospitals, this training also included participants from primary healthcare facilities. During active conflicts, especially in rural and remote areas, primary healthcare facilities may be the first to receive patients during mass casualty incidents. Providing MCM training to healthcare providers at this level strengthens their capacity to triage and stabilize patients prior to transport to larger trauma hospitals and integrates these facilities into the broader national emergency response system. Bringing providers together from multiple healthcare facilities created valuable opportunities for collaboration and allowed participants to share experiences, challenges, and practical solutions for managing MCIs.

Despite the success of this study and lessons learned in conducting MCM training in an active conflict setting, there are a few important limitations to note. Since the sample size was small, the results of this study may not be generalizable to other contexts. This study was also limited by the pre-post evaluation design, in which outcomes were confined to short-term knowledge and self-reported confidence improvements rather than objective performance or patient outcomes during real MCIs. This could be addressed in future studies by including a control group and performing longitudinal follow-up assessments at the facilities of both groups.

While there were benefits to conducting the training in Mogadishu, the centralized training also limited the course instructors’ ability to assess the resources and infrastructure at the health care facilities targeted in the course. Limitations in resources or infrastructure could affect adoption, implementation, and maintenance of the MCM plans developed during the course. Follow-up studies at each facility would offer a more robust understanding of the challenges to MCM readiness and strategies to improve preparedness.

We also encountered language challenges, since some participants had limited English language. The training team sought to address language and contextual barriers by delivering lectures and discussions in both English and Somali. The team was also composed primarily of Somali trainers, allowing for more effective teaching and meaningful discussions about the practical challenges of managing mass casualty incidents in Somalia, but translation of teaching materials into Somali would have made training more accessible.

## Conclusion

The study demonstrates the success of the WHO MCM course in improving knowledge and confidence for frontline healthcare providers at six hospitals and two healthcare facilities in Somalia. Although limited by small sample size and reliance on self-reported outcomes, findings suggest the course may contribute to improved MCM knowledge and confidence in conflict-affected settings. In the future, we hope to implement MCM courses on-site to further enhance preparedness and response capabilities through hospital-based MCI simulations. Future studies should also be conducted to evaluate challenges to adoption and implementation of MCM concepts, as well as the impact of the course on patient outcomes in conflict zones.

## Availability of data and materials

All datasets used and/or analyzed during the current study are available from the corresponding author on reasonable request.

## Dissemination of results

Results from this study were shared with staff members at IMC through a formal report. The results were also presented at AfCEM 2024 in an oral presentation.

## CRediT authorship contribution statement

**Mohamed Mukhtar Ali:** Formal analysis, Investigation, Data curation, Writing – original draft, Writing – review & editing, Software, Visualization, Validation. **Barkhad Mohamed Ismail:** Investigation, Writing – original draft, Writing – review & editing, Visualization, Validation. **Mohamed Hussein:** Project administration, Resources, Writing – review & editing, Supervision, Validation. **Meaghan M. Sydlowski:** Project administration, Investigation, Resources, Validation. **Nichole Michaeli:** Conceptualization, Methodology, Project administration, Formal analysis, Investigation, Data curation, Writing – original draft, Writing – review & editing, Supervision, Software, Visualization, Validation.

## Declaration of competing interest

The authors declare that they have no known competing financial interests or personal relationships that could have appeared to influence the work reported in this paper.
